# Plasmonic property of gold nanoparticles to predict nanozyme activity: towards the colorimetric detection of manganese(ii)

**DOI:** 10.1039/d6ra07418a

**Published:** 2026-09-16

**Authors:** Melisew Tadele Alula, Mildred Lesang Madingwane

**Affiliations:** a Department of Chemical and Forensic Sciences, School of Pure and Applied Sciences, Botswana International University of Science and Technology Plot 10071, Private Bag 16 Palapye Botswana alulam@biust.ac.bw +267-4900102 +267-76126741

## Abstract

Because of its remarkable versatility and indispensable properties, the need for manganese for different applications has grown tremendously. The ever-growing need and the beneficiation of manganese ore, however, have increased the emission of manganese into the environment, such as into water sources, negatively affecting human and animal health. This necessitates a simple method for the analysis of manganese in water resources. The intrinsic peroxidase-like activity of gold nanoparticles (AuNPs) is considered for the detection of Mn^2+^. The peroxidase-like activity of AuNPs using 3,3′,5,5′-tetramethylbenzidine (TMB) as a chromogenic substrate decreased upon the addition of Mn^2+^ to the AuNP nanozyme sensing system. The decrement in the peroxidase-like activity is related with the enlargement of the AuNP size and is dependent on the concentration of Mn^2+^. A linear relationship between absorbance intensity and Mn^2+^ concentration was established for the concentration ranges of 5–60 µM and 60–90 µM. Using the slope and the standard deviation of the linear line for the concentration range of 5–60 µM, the limit of detection (LOD) was determined to be 0.227 µM. The sensing system exhibited excellent selectivity for Mn^2+^. In conclusion, the proposed method opens an avenue for the detection of different target analytes based on the enzyme-like activities of metallic nanoparticles, which could be tuned by aggregation.

## Abstract 2

The intrinsic peroxidase-like activity of gold nanoparticles (AuNPs) is considered for the detection of Mn^2+^.

## Graphical abstract

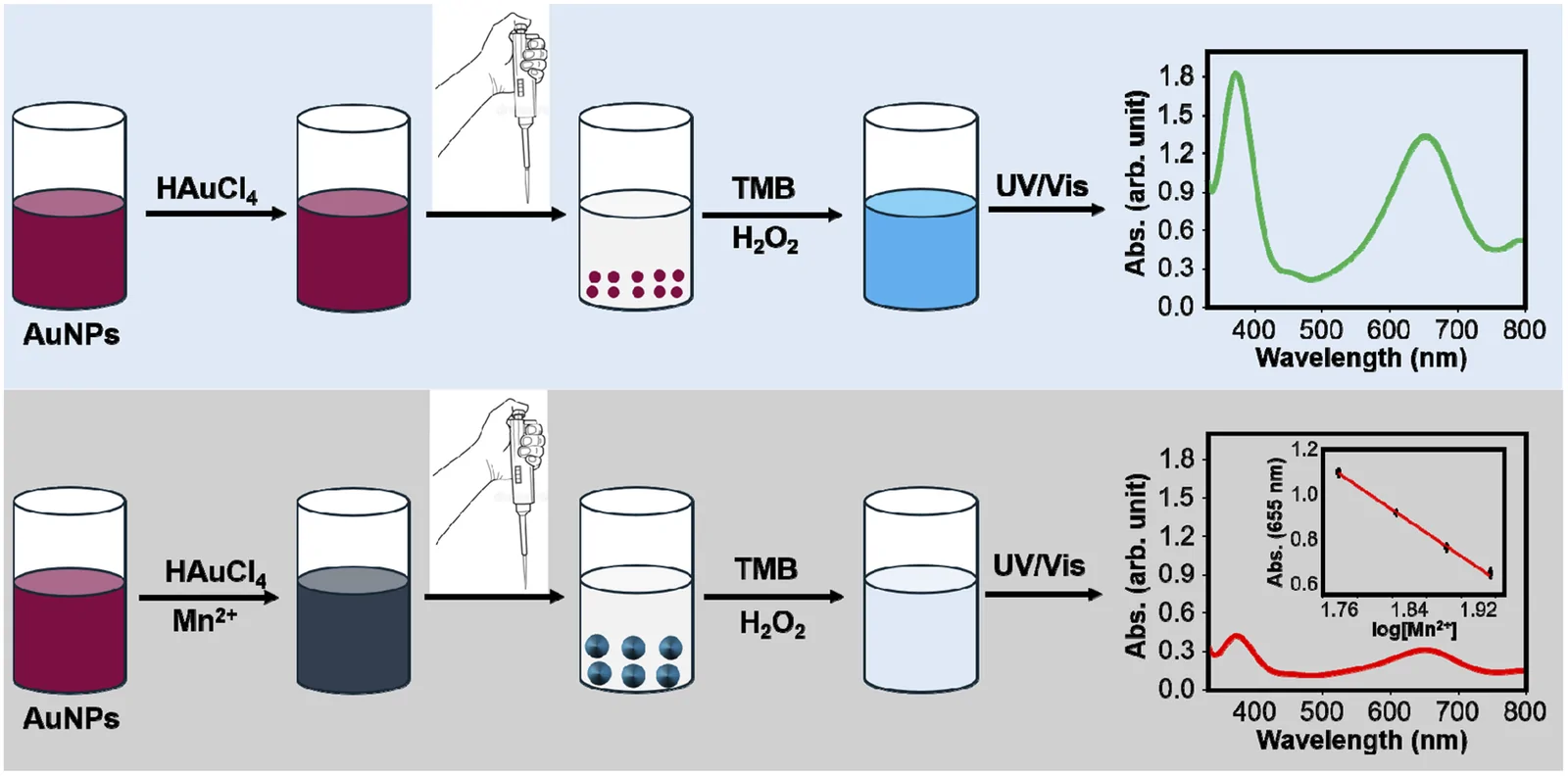

## 1. Introduction

Manganese is widely found in nature and has a wide range of applications. It is intensively used in various products such as batteries, supplements, pesticides, ceramics, fertilizers, gasoline additives, and catalysts.^1,2^ It is an essential element required for living organisms, ranging from bacteria to human beings. Some enzymes require manganese for their formation.^3^ Among the eleven oxidation states of manganese, compounds containing Mn^2+^, Mn^4+^, and Mn^7+^ are biologically and environmentally important.^3^ Because of its remarkable versatility and indispensable properties, the need for manganese in different applications has grown tremendously. This has increased the socio-economic benefits by creating jobs and increasing revenue from mining activities. However, the ever-increasing need and the beneficiation of manganese ore have increased the emission of manganese into the environment, such as into water sources, negatively affecting human and animal health.^4^ It is therefore important to monitor the concentration of Mn^2+^.

Attractive properties such as low cost, rapid signal readout, simplicity, and the requirement of simple instruments have contributed to the utilization of colorimetric methods for the detection of different metal ions. These remarkable features reduce the limitations associated with the utilization of traditional analytical techniques including ion chromatography, mass spectrometry, surface-enhanced Raman spectroscopy, and atomic spectroscopy that are used for the analyses of metal ions.^5,6^ The synthesis of plasmonic metallic nanomaterials to produce readable signals has become a common practice in the analysis of metal ions. Strong optical absorption and scattering of light due to the distinctive localised surface plasmon resonance (LSPR) property of silver and gold nanoparticles (AgNPs/AuNPs) in the visible region of the electromagnetic radiation enable the colorimetry detection of target analytes.^7–9^ These nanoparticles can display higher extinction coefficients than dyes, enabling the easy detection of colour changes that occur when the target analyte interacts with them. The variation in the extent of aggregation and dispersion of the nanoparticles due to target analytes, which could change the plasmonic property, accounts for the sensing of the analytes.^7^ The use of plasmonic nanoparticles for colorimetric detection is not limited to their LSPR property. Like other nanomaterials, plasmonic nanoparticles show the features of nanozymes that could be used for colorimetric detection.^10^

Nanozymes are nanomaterials with enzyme-like activities that can catalyse reactions involving enzyme substrates.^11,12^ Nanozymes catalyse chromogenic reactions to give easy-to-read and sensitive signals for the analyses of various target analytes.^12^ Simple synthesis methods, low cost, and long storage time make nanozyme-based detection systems preferable alternatives to enzyme-based detection systems.^13^ Nanozyme-based detection systems rely on the inhibition or enhancement of the catalytic activity of nanozymes in the presence of the target analyte. For example, the oxidase-like activity of nanoceria was boosted in the presence of fluoride ions. The fluoride ions adsorbed on the surface of nanoceria reduced its surface energy and facilitated electron transfer, resulting in over a hundred-fold improvement in the oxidase-like activity.^14^ The weak peroxidase-like activity of cysteine-coated Fe_3_O_4_ was resumed when Hg^2+^ was added. The active surfaces of Fe_3_O_4_ were exposed when the Hg^2+^-cysteine coordination was formed, leaving more Fe_3_O_4_ surfaces to catalyse the oxidation of TMB for the sensitive detection of Hg^2+^.^15^ Similarly, the enhancement in the enzyme-like activity due to the interaction of mercury with noble metal nanoparticles is due to the change in the surface of the catalysts by amalgamation. This amalgamation may facilitate electron transfer and enhance the catalytic activity for the detection of Hg^2+^.^16–21^ The enzyme-mimicking activity of nanozymes was also enhanced in the presence of Cr^6+^. For example, the faster electron transfer between Ni^2+^ and the Co^2+^/Co^3+^ centres in the nanoporous Ni(OH)_2_/Co_3_O_4_ heterojunction nanostructure improved the formation of reactive oxygen species for the oxidation of TMB. The presence of Cr^6+^ further enhanced the catalyst kinetics for the detection of Cr^6+^.^22^ On the other hand, the enzyme-mimicking activity of nanozymes can be inhibited in the presence of some target analytes. The target analytes may poison the nanozyme^23,24^ or compete with the chromogenic substrate for the hydroxyl free radical, resulting in the inhibition of the catalytic activity. Biomolecules such as cysteine, dopamine, glutathione, and ascorbic acid, which can consume reactive oxygen species (ROS), are usually detected using this strategy.^25–29^

In this study, an analyte-mediated inhibition of the peroxidase-like activity of colloidal AuNPs is reported for the detection of Mn^2+^. The detection system targets the LSPR and catalytic property of AuNPs; the variation in the LSPR property is manifested in the peroxidase-like activity of AuNPs. Upon the addition of Mn^2+^, the LSPR property of AuNPs changes and is implicated in the peroxidase-like activity. Taking this change into consideration, the intrinsic peroxidase-like activity of AuNPs was investigated. Using TMB as a chromogenic substrate, it was found that upon the addition of Mn^2+^, the catalytic oxidation reaction was inhibited. The addition of Mn^2+^ to AuNPs and HAuCl_4_ initiates the growth of AuNPs, producing enlarged AuNPs. The relatively larger size of AuNPs due to the addition Mn^2+^ account for the impediment of the catalytic activity. Remarkably, the peroxidase-like activity of AuNPs upon the addition of Mn^2+^ decreased proportionally with the concentration of Mn^2+^, implying that particle sizes are concentration (Mn^2+^)-dependent. Based on the inhibition of the peroxidase-like activity, a colorimetric method is established for the detection of Mn^2+^. Even though few metal ions, such as Cr^6+^ and Hg^2+^, and ROS-consuming biomolecules, including cysteine, glutathione, dopamine, and ascorbic acid, are detected based on the inhibition of the catalytic activities of nanozymes, the detection of Mn^2+^ based on the inhibition of nanozyme catalytic activity is rare. This study reports the detection of Mn^2+^ based on the inhibition of the catalytic activity of AuNPs.

## 2. Experimental section

### 2.1. Chemicals

Analytical grade chemicals were used as received from the suppliers with no further treatments. Aqueous solutions were prepared using Millipore double deionized water. Gold(iii) chloride trihydrate (HAuCl_4_·3H_2_O), chromium(iii) chloride (CrCl_3_·6H_2_O), silver nitrate (AgNO_3_), glacial acetic acid (CH_3_COOH), anhydrous sodium acetate (CH_3_COONa), iron(iii) chloride hexahydrate (FeCl_3_·6H_2_O), mercury(ii) nitrate (Hg(NO_3_)_2_), cobalt(ii) nitrate hexahydrate (Co(NO_3_)_2_·6H_2_O), manganese(ii) sulfate monohydrate (MnSO_4_·H_2_O), and lead(ii) nitrate (Pb(NO_3_)_2_) were purchased from Sigma-Aldrich. Iron(ii) sulphate heptahydrate (FeSO_4_·7H_2_O), zinc sulphate heptahydrate (ZnSO_4_·7H_2_O), sodium chloride (NaCl), and magnesium nitrate hexahydrate (Mg(NO_3_)_2_·6H_2_O) were obtained from Rochelle chemicals. Trisodium citrate dihydrate (Na_3_C_6_H_5_O_7_·2H_2_O) was purchased from Univar Solutions Inc. Zinc chloride (ZnCl_2_) and calcium nitrate tetrahydrate (Ca(NO_3_)_2_·4H_2_O) were obtained from Merck. Potassium dichromate (K_2_Cr_2_O_7_) and potassium nitrate (KNO_3_) were purchased from Minema Chemicals. Barium chloride dihydrate (BaCl_2_·2H_2_O) was obtained from the Associated Chemical Enterprises. Nickel sulphate hexahydrate (NiSO_4_·6H_2_O) was purchased from Alpha Chemicals.

### 2.2. Preparation of AuNPs

A previously reported method with minor modifications was employed to prepare AuNPs.^6^ In a conical flask, 100 mL of a 0.25 mM HAuCl_4_ aqueous solution was stirred to boil. When it started boiling, 1 mL of a 5% trisodium citrate aqueous solution was added rapidly. The colour of the solution appeared wine-red 20 min after the addition of the citrate solution, indicating the formation of AuNP colloids. The reaction was stopped by removing the flask and placing it in an ice bath.

### 2.3. Instrumentation

The UV/visible extinction and absorption spectra were collected using a Thermo Fisher spectrophotometer. Quartz cuvettes of 1 cm path length were used as sample holders. The TEM images were collected using a Talos F200X G2 transmission electron microscope.

### 2.4. Peroxidase-like activity of AuNPs for the detection of Mn^2+^

**Scheme 1:**
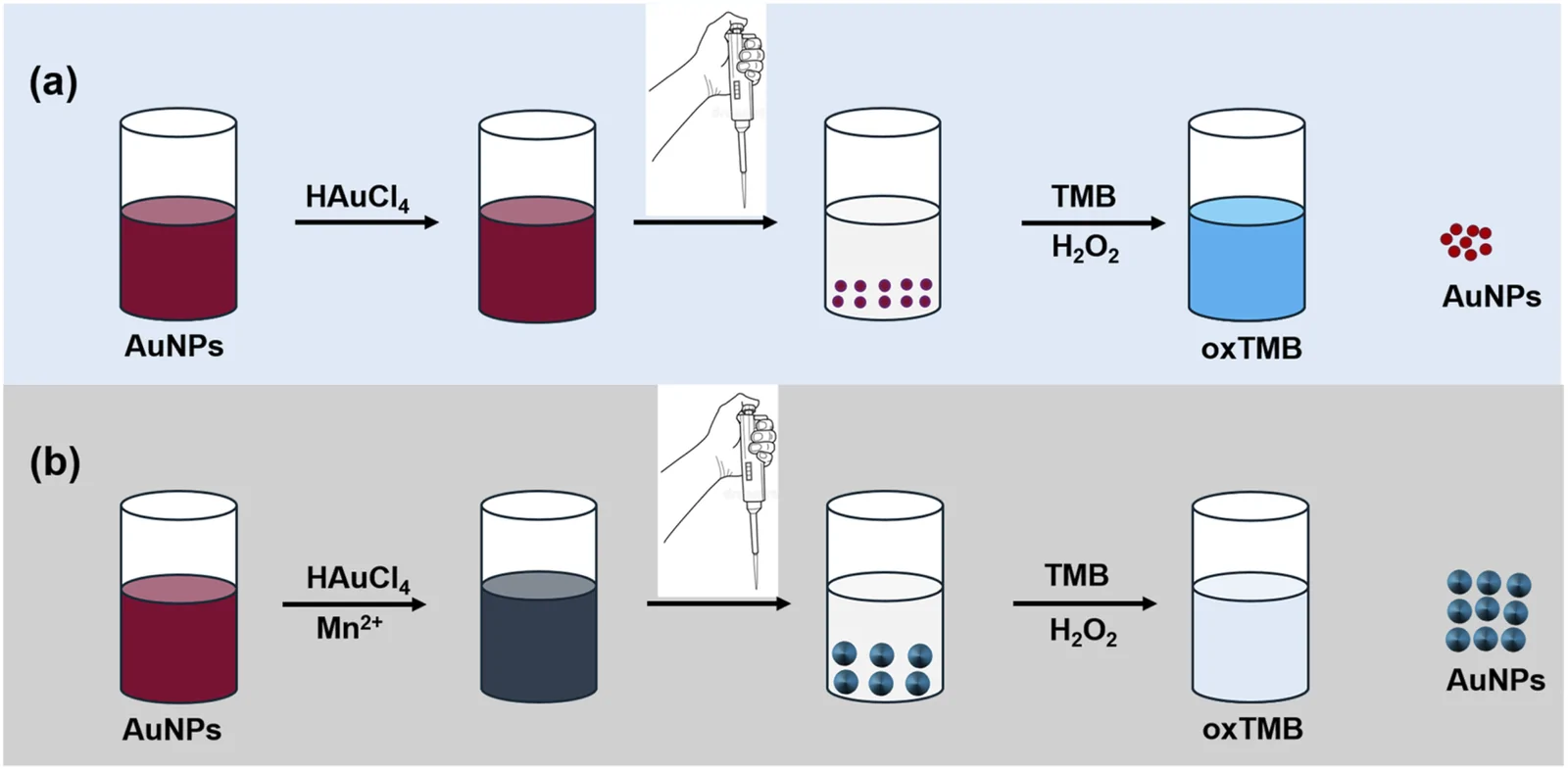
(a) Peroxidase-like activity of AuNPs and (b) peroxidase-like activity of AuNPs for the detection of Mn^2+^.

The detection of Mn^2+^ involved two steps. Initially, in a 2 mL Eppendorf tube, 0.4 mL of AuNP colloids, 0.8 mL of a 0.4 mM HAuCl_4_ solution, and 0.8 mL of Mn^2+^ of different concentrations were incubated for 60 min. From the mixture, 0.5 mL was pipetted and mixed with a nanozyme system comprising an acetate buffer solution (1.4 mL of pH 4.0), 100 µL of TMB (6 mM), and 20 µL of H_2_O_2_ (4.9 M). The mixture was incubated for 10 min before the UV/visible absorption measurements were done. The scheme for the preparation of the nanozyme system and its peroxidase-like activity for the detection of Mn^2+^ is given in Scheme 1.

### 2.5. Determination of Mn^2+^ in borehole ground water

The borehole groundwater was used to evaluate the feasibility of our method for the determination of Mn^2+^ in real samples. A polyvinylidene fluoride filter membrane (0.22 µm) was used to filter the borehole water. The filtered water was diluted ten times, and solutions of different concentrations were prepared by spiking with a solution of Mn^2+^. The same procedure used for the detection of Mn^2+^ in deionized water was followed.

## 3. Results and discussion

### 3.1. Properties of AuNPs

**Fig. 1:**
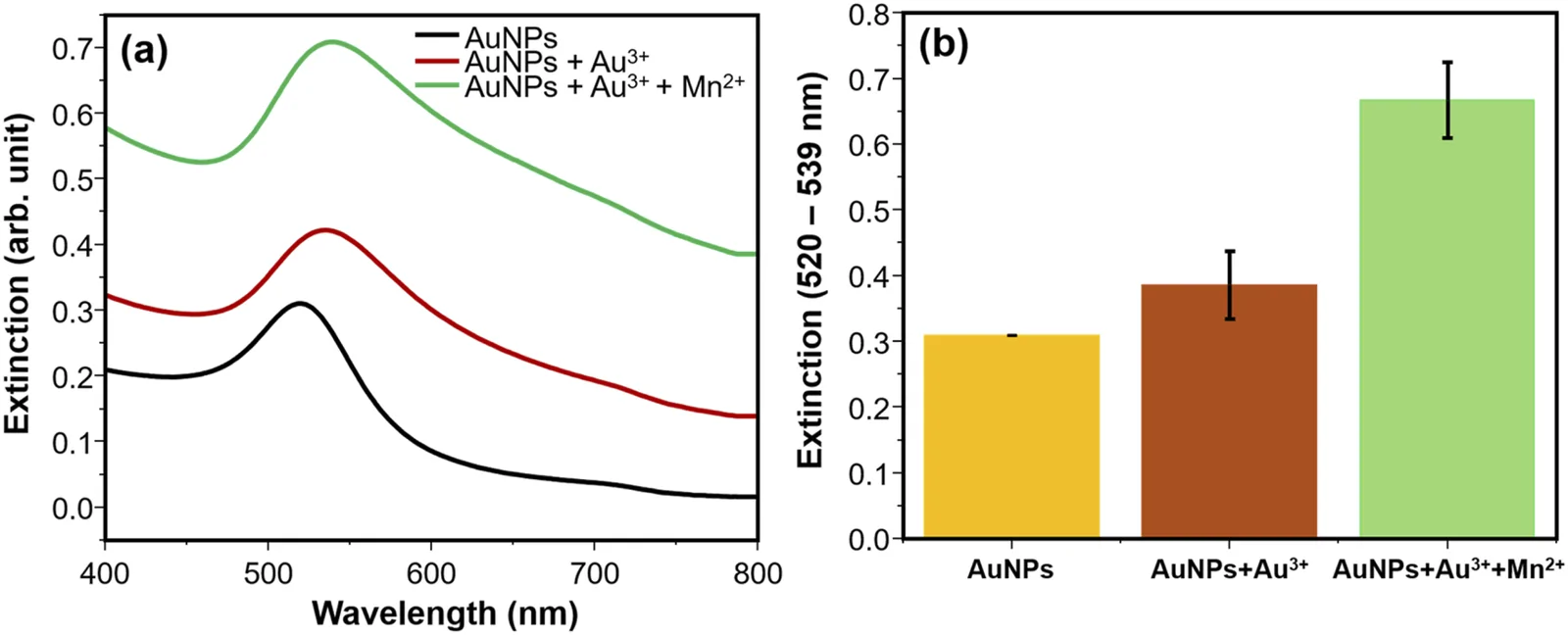
(a) Extinction spectra of AuNPs under different conditions: AuNPs alone, AuNPs + HAuCl_4_, and AuNPs + HAuCl_4_ + Mn^2+^. (b) Increment in the extinction intensities of AuNPs alone, AuNPs + HAuCl_4_, and AuNPs + HAuCl_4_ + Mn^2+^ (*n* = 3).

A simple wet chemical citrate-reduction method was used for the synthesis of AuNPs. The extinction spectra of the as-synthesized AuNPs, HAuCl_4_ + AuNPs, and HAuCl_4_ + AuNPs + Mn^2+^ are given in Fig. 1a. The extinction spectrum with the *λ*_max_ centred at 520 nm is characteristic of AuNPs (black curve). Upon the addition of HAuCl_4_ to AuNPs, the *λ*_max_ redshifted to 530 nm with a slight increment in the extinction intensity (red curve). To the reaction mixture containing HAuCl_4_ and AuNPs, the Mn^2+^ solution was added, and the radical intensity enhancement of the extinction spectrum was accompanied by a broadened, redshifted *λ*_max_, showing the change in the size of AuNPs (green curve). The increment in intensity shows an increment in the extinction coefficient of AuNPs. Fig. 1b shows the increment in the extinction intensities of AuNPs at three different reaction conditions. In our recently published article, we observed the enlargement of AuNP particles in the presence of Mn^2+^.^6^ The nanoparticle growth can be observed from the LSPR peak maximum and extinction intensity.^30^ The LSPR band shift due to nanoparticle growth, however, remains relatively small in spherical nanoparticles.^30^ The effect of Mn^2+^ concentrations on the growth of AuNPs is investigated. The intensity of the extinction spectra of AuNPs increased with the increasing concentration of Mn^2+^, indicating the growth of AuNPs (Fig. S1). In addition to intensity variation, small band shifts were observed upon varying the concentrations of Mn^2+^. The maximum band shifts to longer wavelengths indicate nanoparticle growth.^30^

**Fig. 2:**
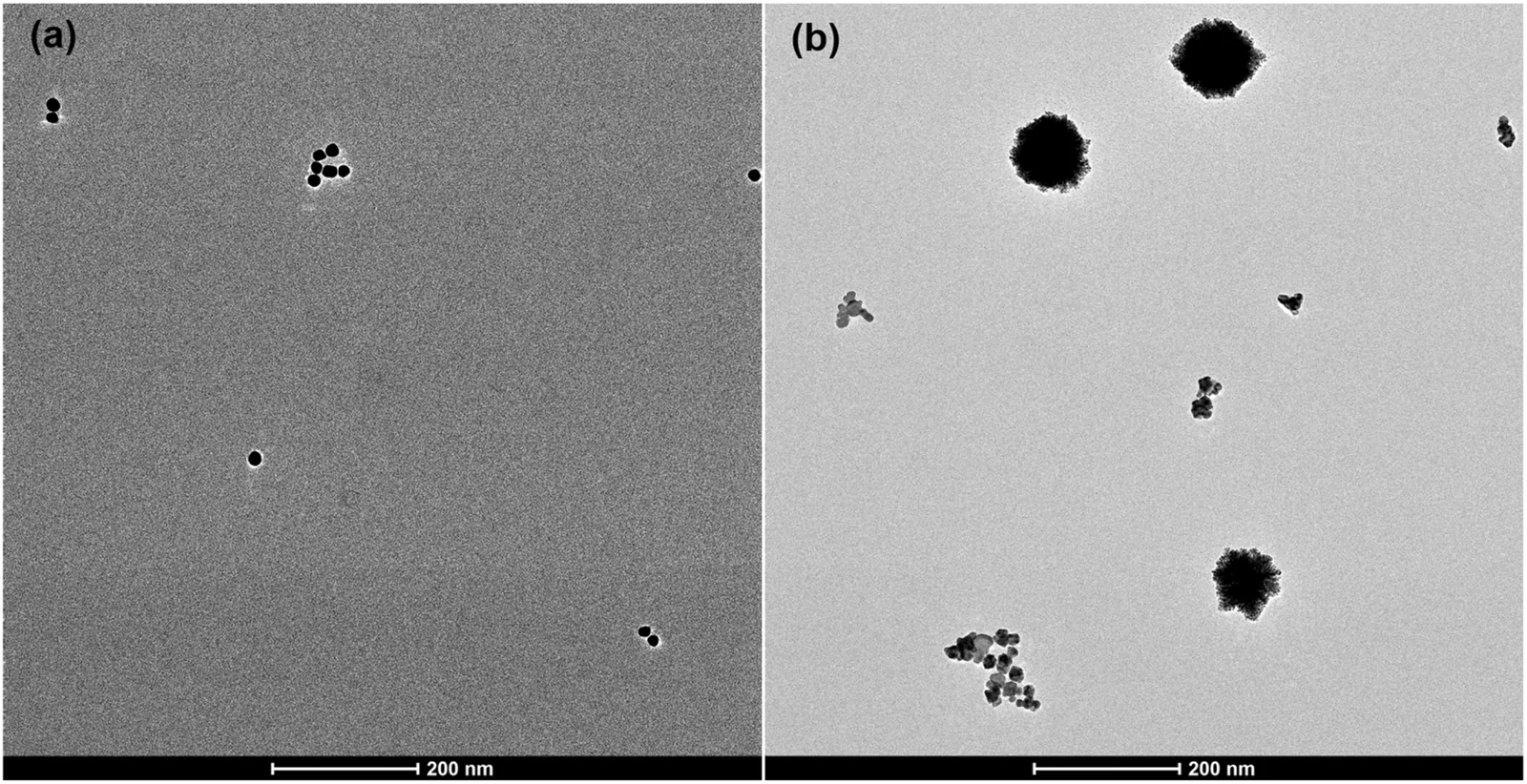
TEM images of AuNPs (a) and AuNPs after the addition of Mn^2+^ (b).

The change in the optical property of AuNPs in response to Mn^2+^ addition is observed from the extinction spectra variation. This observation was supported by the TEM images of the as-prepared AuNPs and AuNPs after the addition of Mn^2+^. The TEM images of AuNPs and AuNPs after the addition of Mn^2+^ are displayed in Fig. 2a. Upon the addition of Mn^2+^, the well-dispersed AuNPs of smaller sizes enlarged and resulted in larger-sized AuNPs (Fig. 2b).

### 3.2. Enzyme-mimicking activity of AuNPs

**Fig. 3:**
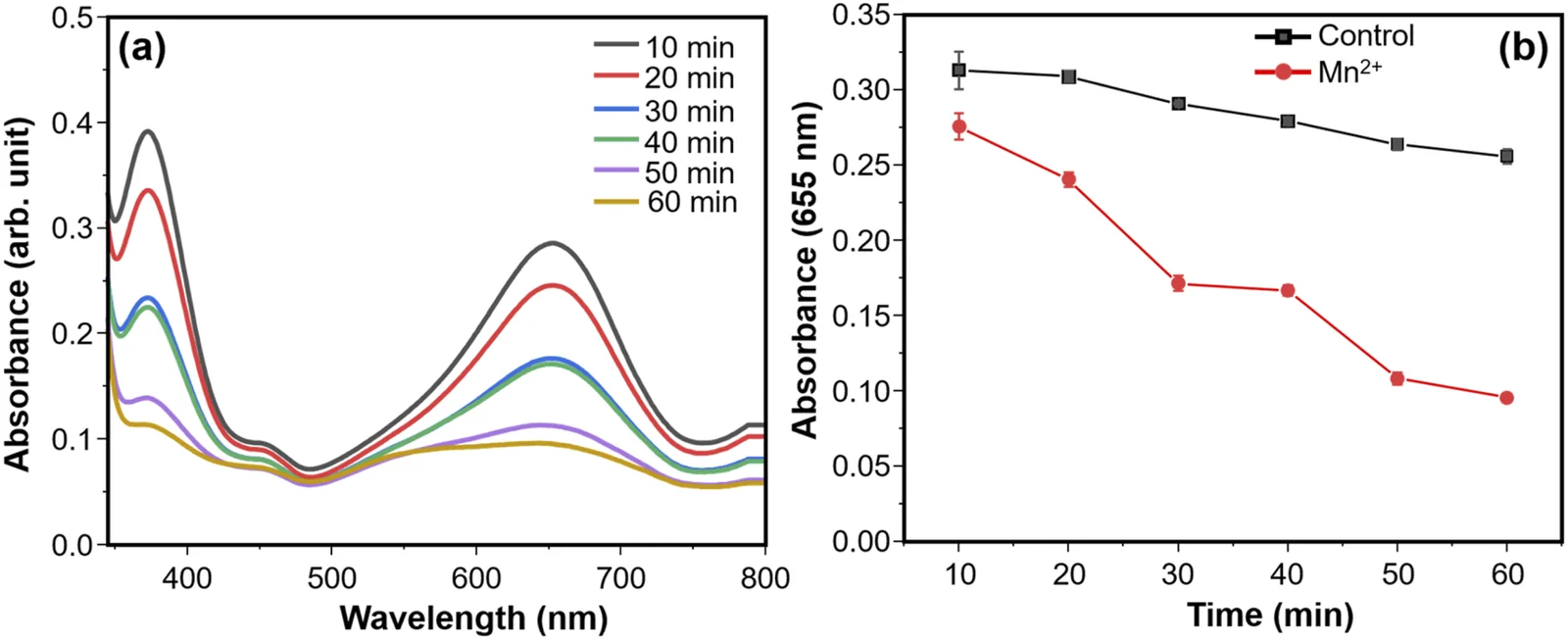
(a) UV/visible absorption spectra of oxTMB. A mixture of AuNPs, HAuCl_4_, and Mn^2+^ was used as nanozymes after they were incubated for different times. (b) Relationship between absorbance intensity and time using AuNPs (black) and AuNPs + Mn^2+^ (red) as nanozymes. The incubation time for the peroxidase-like activity was 10 min (*n* = 3).

AuNPs have been proven to demonstrate intrinsic oxidoreductase activities, resembling the activities of peroxidase, oxidase, catalase, reductase, and superoxide dismutase.^10,31^ The change in the LSPR property of AuNPs in response to Mn^2+^ addition showed a change in the size of AuNPs.^6^ Owing to the fact that the size of nanozymes has a significant effect on the their catalytic efficiency,^32^ the peroxidase-like activity of AuNPs whose sizes changed in response to Mn^2+^ addition was investigated in this study. The difference in the LSPR spectra of the two reaction systems, those with and without Mn^2+^, was evaluated. The first reaction system contained 400 µL of AuNPs, 800 µL of HAuCl_4_ (0.4 mM), and 800 µL of H_2_O (control), while for the second reaction system, 800 µL of Mn^2+^ (100 µM) was used in place of H_2_O. They were incubated for different periods ranging from 10 to 60 min. Every 10 min, 100 µL of reaction mixtures were pipetted and used as nanozymes for the catalytic oxidation of TMB. The pipetted 100 µL from each reaction mixture was mixed with acetate buffer, TMB, and H_2_O_2_ and incubated for 10 min before conducting UV/visible absorption spectroscopy measurements. The absorbance intensity of oxTMB was used to assess the activity. For both systems, the control and Mn^2+^-incorporated systems, the peroxidase-like activity decreased with incubation time, as observed from the oxTMB spectra (Fig. 3a and S2). The decrement is highly pronounced for the reaction system containing Mn^2+^ (Fig. 3b). The decrement in catalytic activity can be ascribed to the enlargement of the sizes of AuNPs upon the addition of HAuCl_4_ and Mn^2+^. Nanozymes of the same material can exhibit varying enzyme activities and catalytic types because of the variations in their morphologies, valence states, and sizes.^33^ For example, smaller-sized Fe_3_O_4_ nanoparticles exhibited higher peroxidase-like activity than Fe_3_O_4_ of larger sizes.^34^ Increasing the size of the particles reduces the density of the active sites in catalysts, consequently decreasing the catalytic activity. For AuNPs, their catalytic activity is size dependent, and any activity variation can be explained by the variation in the adsorption free energies of the substrate and product.^10^ The stability of AuNPs upon storage was also investigated. It was found that no significant variation was observed in the peroxidase-like activity of AuNPs when measured for four consecutive days, indicating the stability of our nanozymes (Fig. S3).

### 3.3. Factors affecting the peroxidase-like activity of AuNPs

**Fig. 4:**
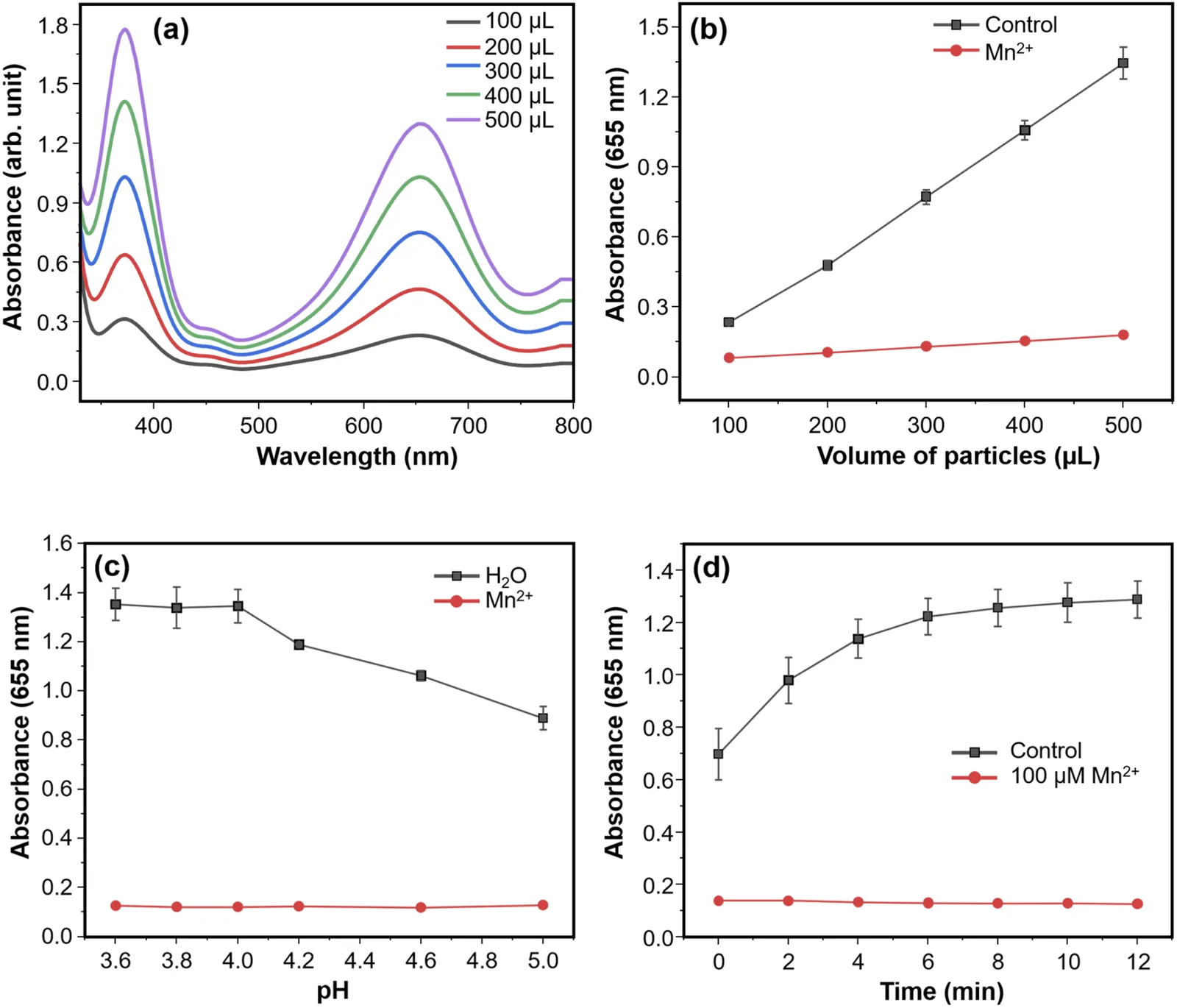
(a) UV/visible absorption spectra of TMB. Different volumes (100–500 µL) of a mixture of AuNPs and HAuCl_4_ were used for the peroxidase-like reaction system after they were incubated for 60 min. (b) Absorbance intensity of oxTMB against the volume of AuNPs for the control (black curve) and Mn^2+^ (100 µM, red curve) reaction systems. (c) Effect of pH on the absorbance intensity of oxTMB with and without Mn^2+^. (d) Effect of time for the catalytic oxidation of TMB for the control (black curve) and Mn^2+^ (red curve, 100 µM) reaction systems (*n* = 3).

Different factors, such as the amount of particles, time, and pH, that affect the catalytic activity were assessed for developing the optimum sensing system. After incubating AuNPs, HAuCl_4_, and Mn^2+^ (or H_2_O as control) for 60 min, different volumes of the mixture were pipetted and used as nanozymes to catalyse the oxidation of TMB. In the absence of Mn^2+^ (control), the catalytic activity increased with the amount of AuNPs. Fig. 4a shows the UV/visible absorption spectra of oxTMB when different volumes of AuNPs were used for the catalytic oxidation systems. The absorbance of oxTMB increased, indicating that more TMB molecules are oxidized as the volume of nanozymes is increased. For the reaction system containing Mn^2+^, no peroxidase-like activity was observed regardless of the amount of AuNPs (Fig. S4). The plot of absorbance intensity against the volume of AuNPs for the control and Mn^2+^ reaction systems is given in Fig. 4b. This shows that the peroxidase-like activity of AuNPs is inhibited by Mn^2+^. The formation of large-sized AuNPs due to the addition of Mn^2+^ accounts for the inhibition of the peroxidase-like activity.

The efficient oxidation of the chromogenic substrates, observed as a change from a colorless to a colourful solution, is highly dependent on the pH of the buffer used in the reaction system.^35^ This is associated with the protonation/deprotonation of the chromogenic substrates in different buffers. For example, *o*-phenylenediamine works efficiently in a phosphate buffer of acidic pH ranging from 6.2 to 7.0.^36^ The two pKa values of the protonated forms of the oxidized product, 2,3-diaminophenazine, account for the variation.^37^ On the other hand, the oxidation of TMB results in the protonation of the amino groups, which depends on the pH of the buffer.^35^ The high catalytic oxidation of TMB is shown in acidic buffer solutions due to the protonation of the amino group.^35,38,39^ Acetate buffers of different pH values ranging from 3.6 to 5.0 were used to assess the effect of pH on nanozyme performance. For the control, similar catalytic activities were observed at the pH values of 3.6, 3.8, and 4.0. A further increment in pH resulted in a gradual decrease in the activity. However, the catalytic performance remained high for higher pH values, showing a relatively wider pH tolerance of the nanozyme system (Fig. 4c). Regardless of the pH of the buffers, no oxidation of TMB was observed for the Mn^2+^ systems, indicating the inhibitory effect of Mn^2+^ on the enzyme-mimicking activity of AuNPs. The effect of reaction time was also studied. The oxidation of TMB increased gradually with time (Fig. 4d). After 6 min, however, no noticeable difference was observed with time, indicating that the reaction was fast. For Mn^2+^ system, no oxidation reaction was observed for all reaction times. The spectra for the control and Mn^2+^ systems are displayed in Fig. S5. A reaction time of 10 min was used for the sensing system.

### 3.4. Detection of Mn^2+^

Considering the catalytic-based colorimetric detection, the target analyte may have an effect on the catalytic performance of nanozymes. The increase or decrease in the catalytic activity influences the signal intensity. The modulation of the catalytic activity can be associated with the dispersion or aggregation of nanozymes, which affects the active catalytic surfaces.^12^ For example, the peroxidase- and glucose-like activities of AuNPs were enhanced in the presence of dopamine, ascribed to the formation of branched gold superstructures.^40^ Similarly, the peroxidase-like activity of gold nanoclusters increased due to aggregation that was induced by Pb^2+^.^41^ On the other hand, the peroxidase-like activity of the bovine serum albumin-protected Au clusters was inhibited in the presence of Hg^2+^. The concentration-dependent inhibition of the peroxidase-like activity enabled the colorimetric detection of Hg^2+^.^42^ Inspired by these results and the change in the LSPR of AuNPs upon the addition of Mn^2+^ that induced enlargement of AuNPs,^6^ we propose an Mn^2+^-mediated growth of AuNPs as a nanozyme-based sensing system for the detection of Mn^2+^. It is found that AuNPs with and without Mn^2+^ show different catalytic performances. Based on this, a sensing system for the detection of Mn^2+^ is established. Thus, the catalytic activity of AuNPs in response to Mn^2+^ was assessed. Initially, AuNPs were mixed with different concentrations of Mn^2+^. 500 µL of the reaction mixtures were pipetted and used as nanozymes. A reaction system composed of TMB, H_2_O_2_, acetate buffer, and a mixture of AuNPs and Mn^2+^ was incubated for 10 min, and UV/visible absorption spectroscopy measurements were made. The catalytic activity decreased as the concentration of Mn^2+^ increased. This can be ascribed to the enlargement of AuNPs with increasing concentrations of Mn^2+^.^6^ The higher catalytic efficiency demonstrated by smaller-sized nanozymes is due to their larger specific surface area.^32^ As the particles grow, the specific surface area of the catalyst that should be accessed by the substrates decreases, resulting in the inhibition of the catalytic activity.

**Fig. 5:**
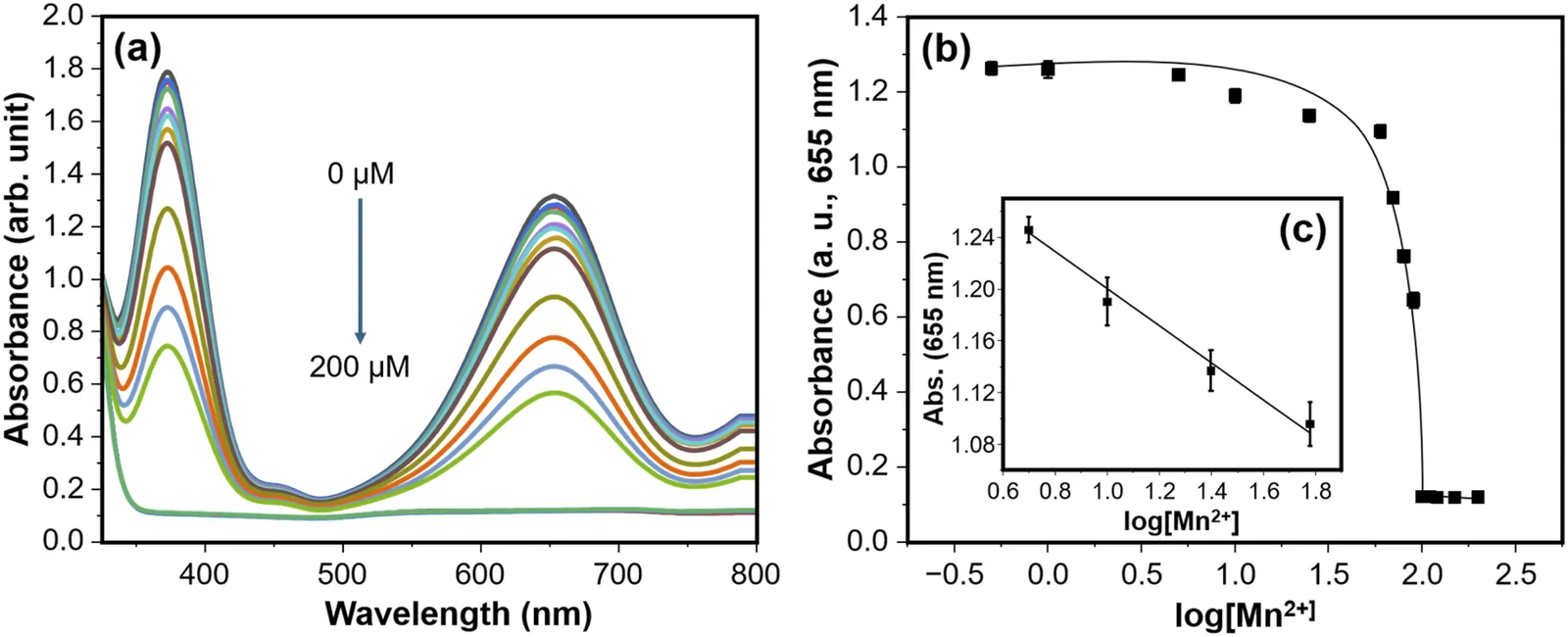
(a) Changes in the absorbance spectra of oxTMB in response to the concentration of Mn^2+^. (b) Concentration profile curve of Mn^2+^ showing the relationship between the absorbance intensity of oxTMB and log[Mn^2+^]. (c) Calibration curve in the concentration range of 5–60 µM (*n* = 3).

The absorption spectra of oxTMB for the sensing system when different concentrations of Mn^2+^ were mixed are displayed in Fig. 5a. The absorption intensity decreased with increasing concentrations of Mn^2+^, indicating that the catalytic activity is concentration (of Mn^2+^) dependent. Therefore, the Mn^2+^ concentration-dependent inhibition of the peroxidase-like activity of AuNPs provides a detection system for the analysis of Mn^2+^. The concentration–profile curve relating the absorbance (of oxTMB) and log[Mn^2+^] is given in Fig. 5b. From this profile curve, two linear curves ranging from 5–60 µM and 60–90 µM are obtained that show the linear relation of concentration and log[Mn^2+^] (Fig. 5c displays the first linear curve). In the concentration range of 5–60 µM, the LOD was computed and found to be 0.227 µM. Our newly developed nanozyme-based colorimetric detection method is compared with the previously reported methods in Table 1. The wider linear range and the low LOD show that the performance of our method is comparable with that of the reported colorimetric methods.

**Table 1:** LOD and linear range of our method and previously reported methods for the colorimetric detection of Mn^2+^

No	Nanomaterial (colorimetric)	Limit of detection (µM)	Linear range (µM)	Reference
1	SAA-DTC-AgNPs	1.7	5–50	43
2	SiNPs	0.532	0.5–6	44
3	4-MBA-MA-AgNPs	0.005	0.5–10	45
4	β-CD-ADM AgNPs	0.5	Not given	46
5	AgNPs	0.2	0.2–2.5	47
6	PVP-AgNPs	0.282	20–80	48
7	AuNPs	0.227	5–60; 60–90	This work

### 3.5. Selectivity of the method

**Fig. 6:**
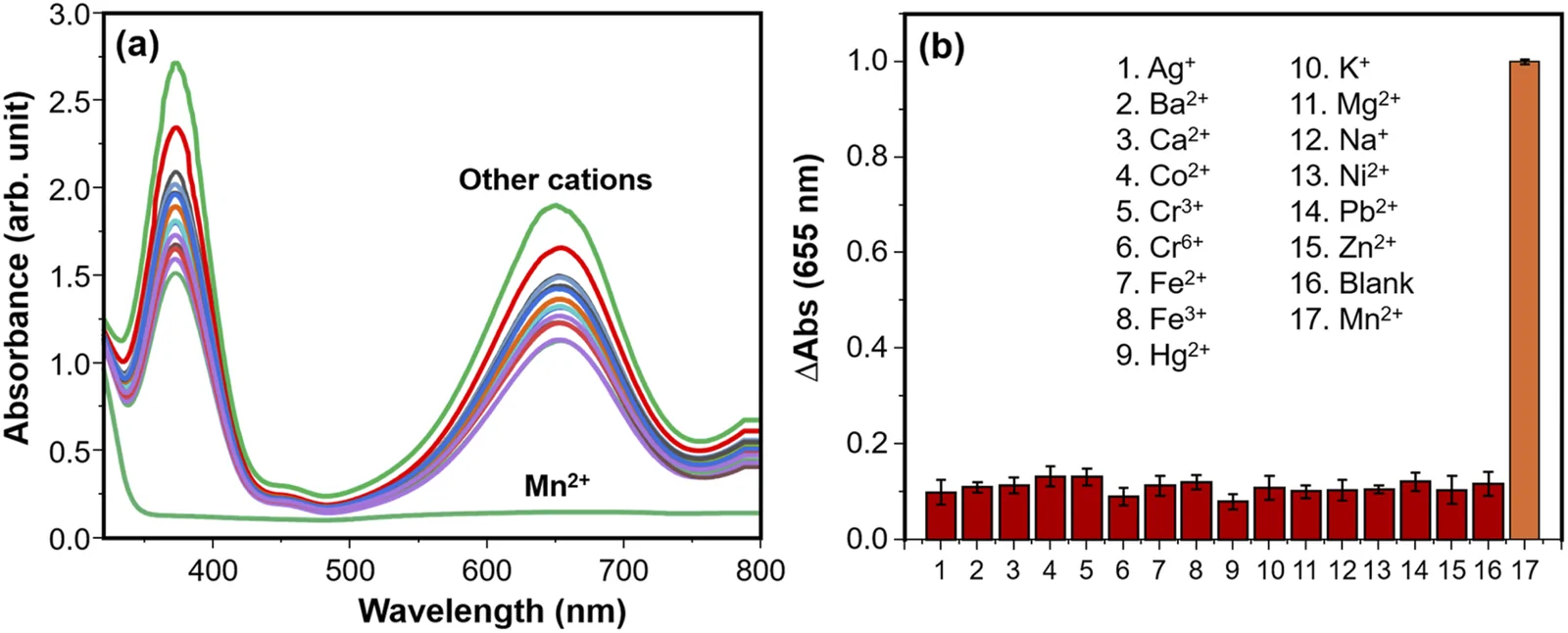
(a) Absorption spectra of oxTMB using AuNPs in the presence of 100 µM cations. (b) Change in the absorbance intensity of oxTMB using AuNPs as nanozymes in the presence of 100 µM cations individually (*n* = 3).

The selectivity of the method was also studied. The same procedure used for the detection of Mn^2+^ was applied for the tested cations. It was found that only Mn^2+^ inhibited the peroxidase-like activity, showing that AuNPs are not affected by other cations (Fig. 6a). This agrees with our previously reported findings.^6^ The bar graph shows the ΔAbs value for each cation (Fig. 6b), where ΔAbs refers to Abs_Mn_^2+^/Abs_cation_. Although many sensing systems based on nanozymes are successful in detecting limited cations such as Hg^2+^, Cr^6+^, Ag^+^, and Pb^2+^,^12^ because of their interactions with nanozymes, our nanozymes can selectively detect Mn^2+^. This unusual response of Mn^2+^ to AuNPs can be ascribed to the enlargement of AuNPs upon the addition of Mn^2+^, which changes the catalytic activity of AuNPs. The enlargement of AuNPs upon the addition of Mn^2+^ can be ascribed to the reduction potential difference between Mn^2+^ and Au^3+^. The high potential differences in the reduction potentials of Mn^2+^ (−1.18 V) and Au^3+^ (+1.52 V) can favour the reduction of Au^3+^ to Au^0^.^9,49^ A similar observation is reported for the reduction of Hg^2+^ to Hg^0^ using Mn^2+^.^50^ The enlargement of AuNPs suppresses the peroxidase-like activity of AuNPs.

### 3.6. Analysis of Mn^2+^ in borehole water

The feasibility of the method for the determination of Mn^2+^ in real samples was tested using borehole ground water. The optimum conditions were used to prepare the sensing system for Mn^2+^-spiked solutions. The absorbance spectra of the resulting oxTMB solutions were collected, and the concentrations of Mn^2+^ were computed from the calibration curve. Recovery rates in the range of 91.2–96.5% were obtained, indicating the high accuracy level of the method. Table 2 shows the recovery rates, relative standard deviation (RSD) and the concentrations of Mn^2+^ obtained by our method using ground borehole water samples.

**Table 2:** Determination of Mn^2+^ in borehole ground water samples

Borehole	Added Mn^2+^ (µM)	Obtained Mn^2+^ (µM)	Recovery (%)	RSD (%) water
Sample 1	5.00	4.79	95.5	1.00
Sample 2	10.00	9.12	91.2	0.98
Sample 3	60.00	57.90	96.5	2.58

## 4. Conclusion

A simple colorimetric method based on the peroxidase-like activity of AuNPs is established. In the presence of HAuCl_4_, the small-sized AuNPs grow upon the addition of Mn^2+^ and resulted in a decrease in the intrinsic peroxidase-like activity of AuNPs. The peroxidase-like activity decreased proportionally with the concentration of Mn^2+^. The method shows excellent selectivity towards Mn^2+^ detection. In contrast to the aggregation-based LSPR approach, our method avoids false-positive outcomes that might arise due to the self-aggregation of colloidal nanoparticles. Importantly, our method is simple that does not require time-consuming modification processes, unlike the aggregation-based LSPR methods. This strategy opens opportunities for aggregation-based colloidal nanozymes for the detection of target analytes. Target analytes that could interact and induce the aggregation of colloidal nanozymes can be detected using this strategy. However, unlike solid nanozymes, it is impractical to separate colloidal nanozymes from the reaction systems and reuse them.

## Author contributions

Melisew Tadele Alula: conceptualization, formal analysis, funding acquisition, methodology, visualization, supervision, validation, writing – original draft, and writing – review & editing. Mildred Lesang Madingwane: investigation and methodology.

## Conflicts of interest

The authors declare that there are no conflicts of interest.

## Supplementary Material

RA-OLF-D6RA07418A-s001

## Data Availability

The data supporting this article have been included as part of the supplementary information (SI). Supplementary information is available. See DOI: https://doi.org/10.1039/d6ra07418a.
