# Supplementary material for: Plasmonic property of gold nanoparticles to predict nanozyme activity: towards the colorimetric detection of manganese(ii)
Source: RSC Adv. 2026 Sep 16;16(44):55409–18. doi: 10.1039/d6ra07418a (PMC13579522; doi:10.1039/d6ra07418a)
Supplement: Supplementary file 1 [file RA-016-D6RA07418A-s001.pdf]

Supplementary Information (SI) for RSC Advances.
This journal is © The Royal Society of Chemistry 2026

Plasmonic Property of Gold Nanoparticles to Predict Nanozyme Activity: Towards Colorimetric Detection of Manganese(II)

Melisew Tadele Alula*, Mildred Lesang Madingwane

Department of Chemical and Forensic Sciences, School of Pure and Applied Sciences, Botswana International University of Science and Technology, Plot 10071, Private Bag 16, Palapye, Botswana. E-mail: alulam@biust.ac.bw; Fax: +267-4900102; Tel: +267-76126741

Supplementary information

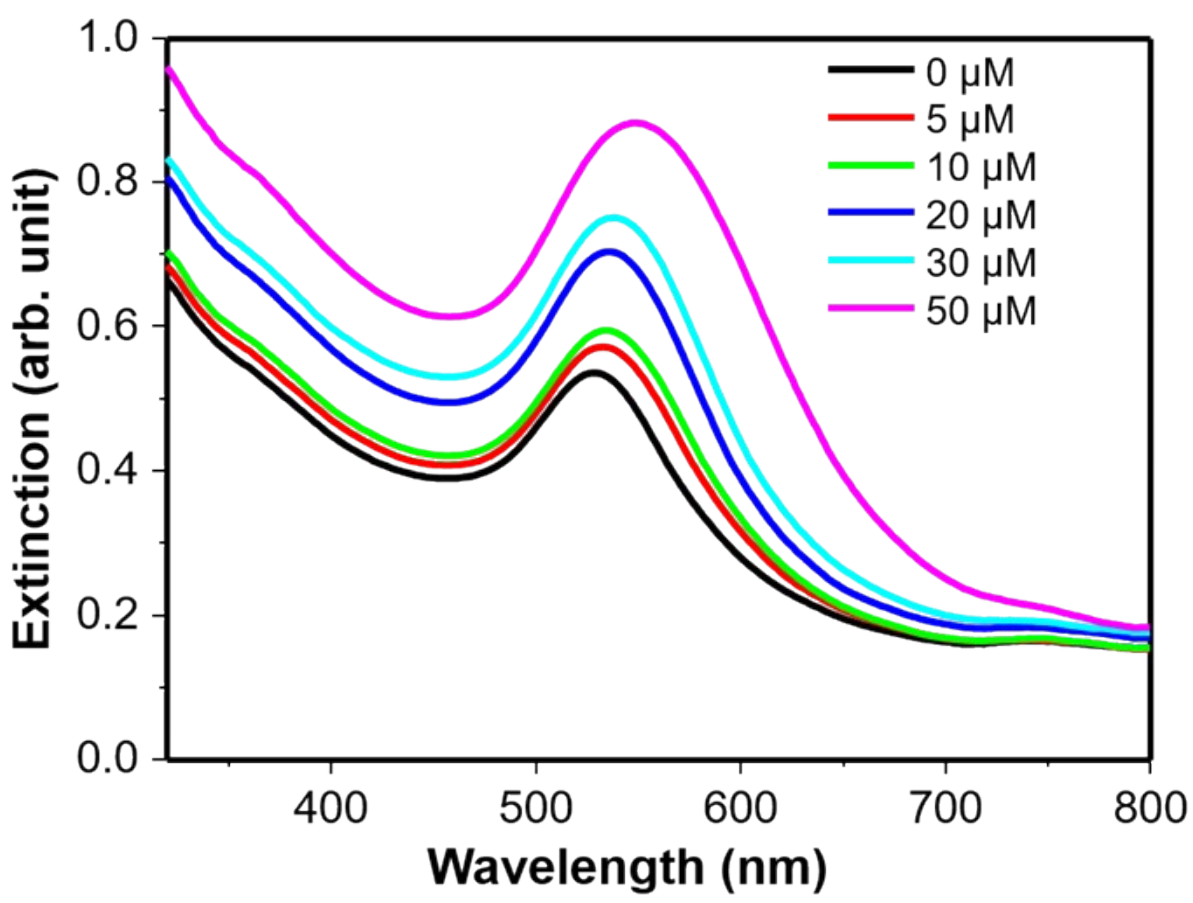

Fig. S1 Extinction spectra of AuNPs. Different concentrations of Mn^{2+} were incubated with AuNPs and $HAuCl_4$ for 10 min.

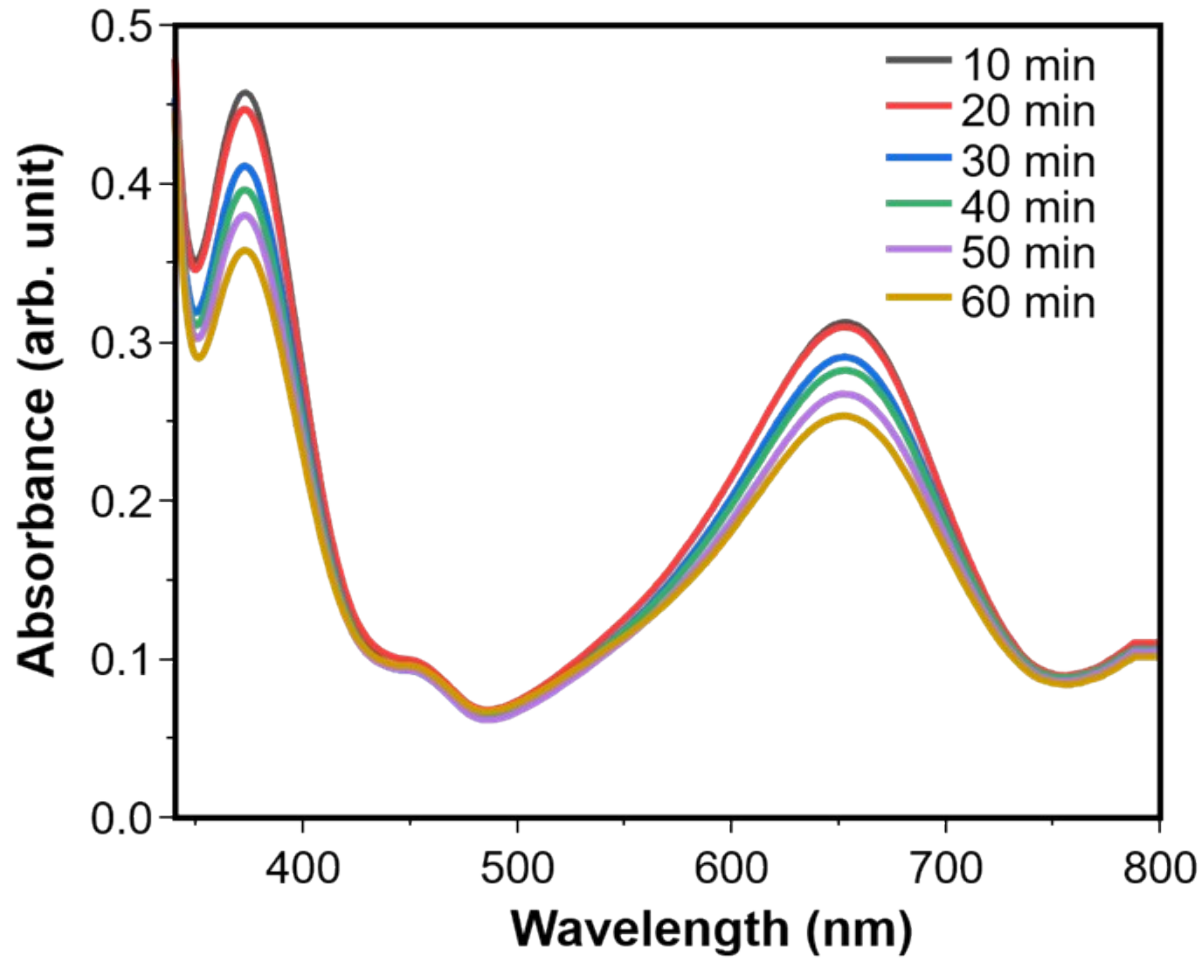

Fig. S2 UV/Visible absorption spectra of oxTMB. A mixture of AuNPs and $HAuCl_4$ was used as nanozymes after they were incubated for different time.

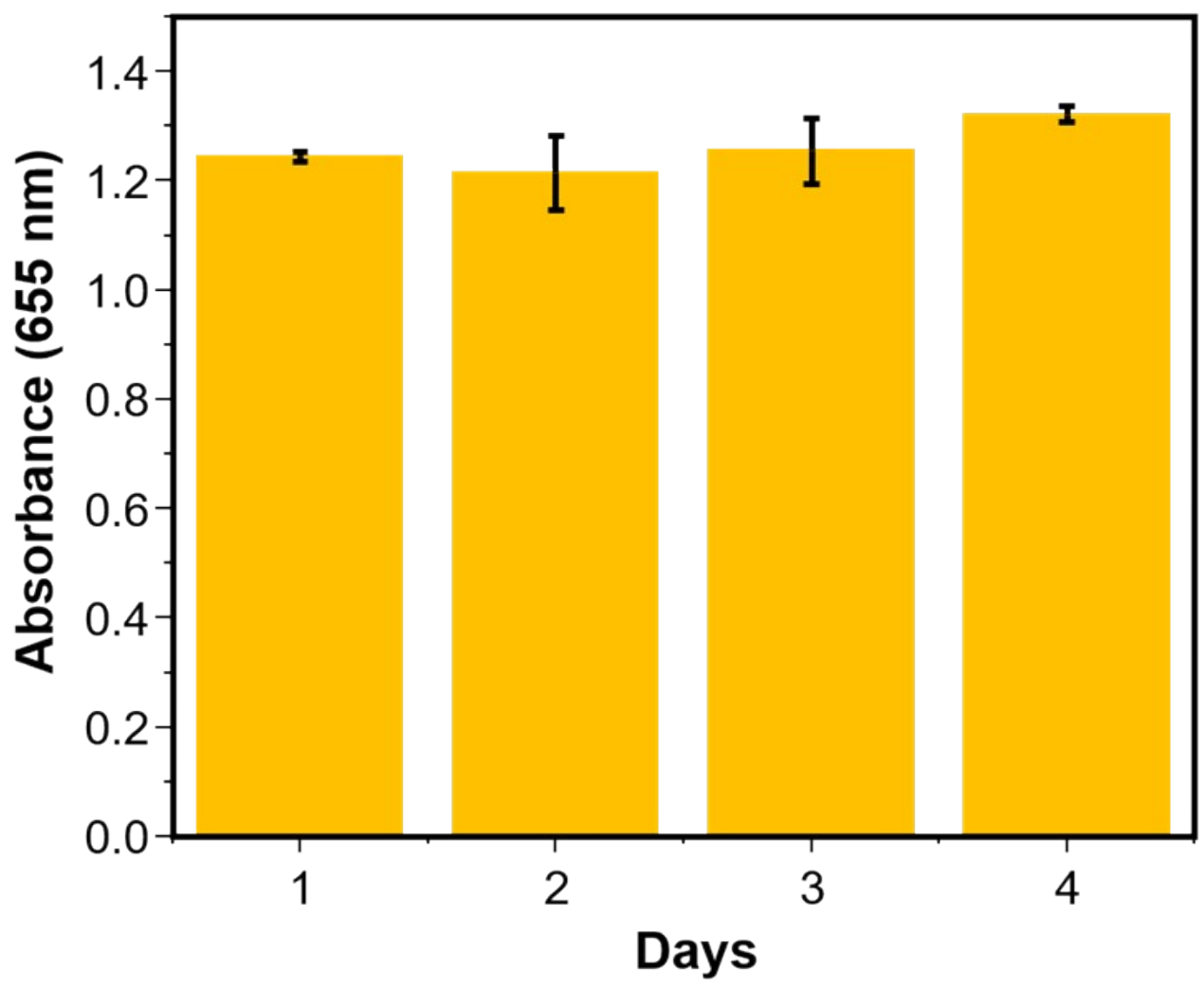

Fig. S3 UV/Visible absorption spectra intensities of oxTMB using AuNPs as nanozymes measured at different days showing the stability of AuNPs.

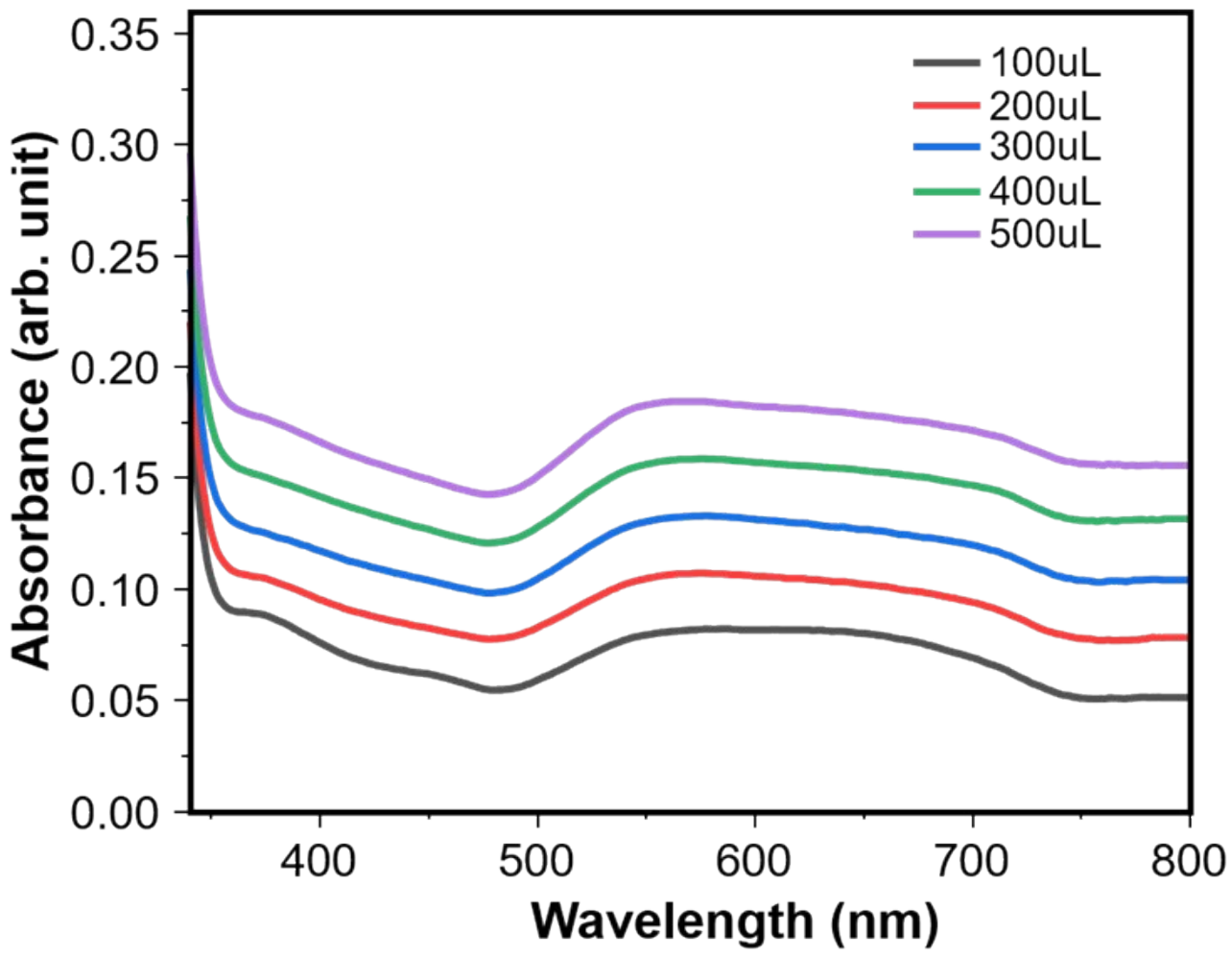

Fig. S4 UV/Visible absorption spectra of TMB. Different volumes (100 – 500 μL) of a mixture of AuNPs, $HAuCl_4$, and Mn^{2+} (100 μM) were used for the peroxidase-like reaction system after they were incubated for 60 min.

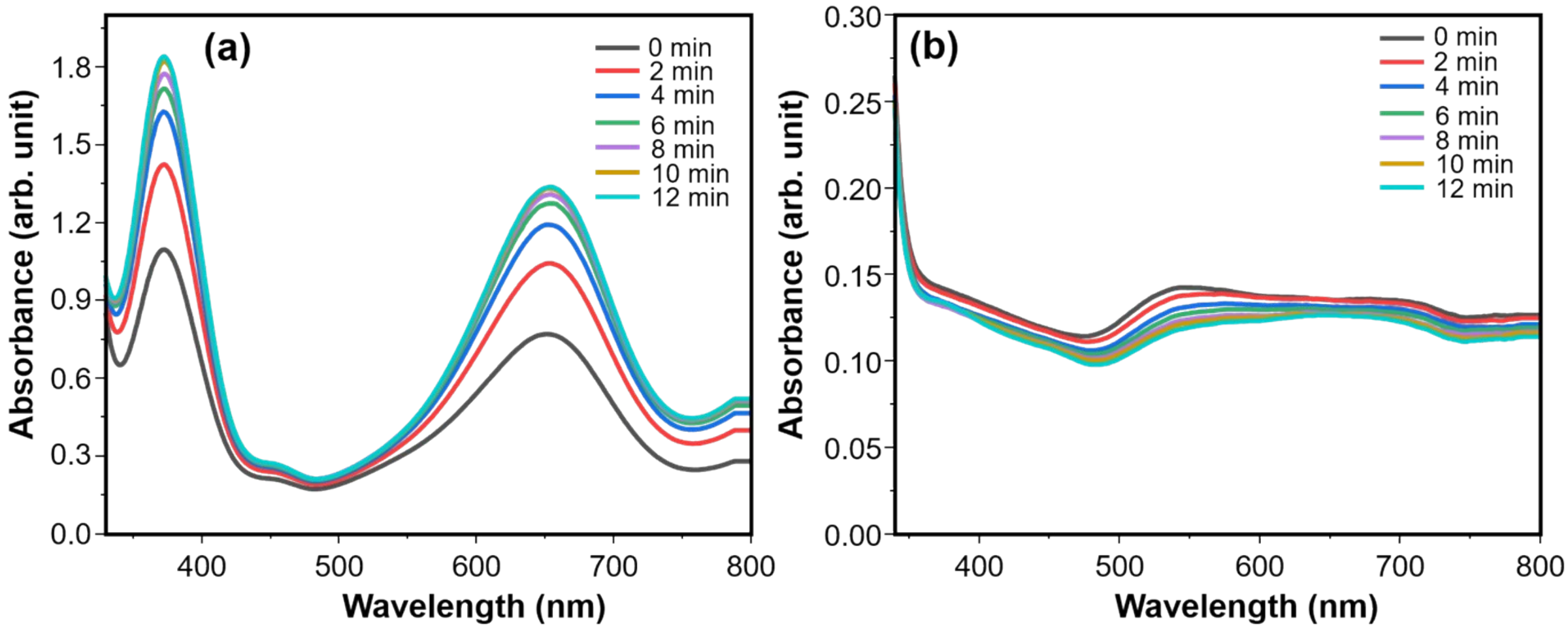

Fig. S5 (a) UV/Visible absorption spectra of oxTMB for the control reaction systems in a range of 0 to 12 min. (b) UV/Visible absorption spectra of oxTMB for the Mn^{2+} (100 μM) reaction systems in a range of 0 to 12 min. A 500 μL mixture of AuNPs, $HAuCl_4$, and Mn^{2+} (100 μM) or H_2O (control) were used for the peroxidase-like reaction system after they were incubated for 60 min.
